# HDAC6/HNF4α loop mediated by miR-1 promotes bile acids-induced gastric intestinal metaplasia

**DOI:** 10.1007/s10120-020-01108-x

**Published:** 2020-07-23

**Authors:** Na Wang, Min Chen, Zhen Ni, Ting Li, Jiaoxia Zeng, Guofang Lu, Jiaojiao Wang, Jian Zhang, Siran Wu, Yongquan Shi

**Affiliations:** 1 State Key Laboratory of Cancer Biology, Xijing Hospital of Digestive Diseases, The Fourth Military Medical University, No. 15 West Changle Road, Xi’an, 710032 Shaanxi China; 2 Department of Gastroenterology, General Hospital of Western Theater Command, Chengdu, Sichuan China; 3 Department of Cardiovascular Medicine, First Affiliated Hospital of Medical School, Xi’an Jiaotong University, Xi’an, China; 4 Key Laboratory of Environment and Genes Related to Diseases, Xi’an Jiaotong University, Ministry of education, Xi’an, China; 5 Shannxi University of Chinese Medicine, Xi’an, China

**Keywords:** Gastric intestinal metaplasia, HDAC6, HNF4α, CDX2, Gastric cancer

## Abstract

**Background:**

Gastric intestinal metaplasia (IM) is considered a precancerous lesion, and bile acids (BA) play a critical role in the induction of IM. Ectopic expression of HNF4α was observed in a BA-induced IM cell model. However, the mechanisms underlying the upregulation of the protein in IM cells remains to be elucidated.

**Methods:**

The effects of HNF4α on gastric mucosal cells in vivo were identified by a transgenic mouse model and RNA-seq was used to screen downstream targets of deoxycholic acid (DCA). The expression of pivotal molecules and miR-1 was detected by immunohistochemistry and in situ hybridization in normal, gastritis and IM tissue slides or microarrays. The transcriptional regulation of HDAC6 was investigated by chromatin immunoprecipitation (ChIP) and luciferase reporter assays.

**Results:**

The transgenic mouse model validated that HNF4α stimulated the HDAC6 expression and mucin secretion in gastric mucosa. Increased HDAC6 and HNF4α expression was also detected in the gastric IM cell model and patient specimens. HNF4α could bind to and activate HDAC6 promoter. In turn, HDAC6 enhanced the HNF4α protein level in GES-1 cells. Furthermore, miR-1 suppressed the expression of downstream intestinal markers by targeting HDAC6 and HNF4α.

**Conclusions:**

Our findings show that the HDAC6/HNF4α loop regulated by miR-1 plays a critical role in gastric IM. Blocking the activation of this loop could be a potential approach to preventing BA-induced gastric IM or even gastric cancer (GC).

**Electronic supplementary material:**

The online version of this article (10.1007/s10120-020-01108-x) contains supplementary material, which is available to authorized users.

## Introduction

Gastric cancer (GC) is the third leading cause of cancer-related death worldwide [1]. Universally, the occurrence and stepwise development of intestinal gastric cancer (IGC) follow the Correa model: superficial gastritis–atrophic gastritis–metaplasia–hyperplasia–cancer [2, 3]. Early recognition and prevention of precancerous lesions may provide potential opportunities to prevent the appearance of GC.

Although a large number of studies have confirmed that intestinal metaplasia (IM) increases the risk of GC [4–7], the mechanisms underlying the initiation and progression of IM are poorly understood. In general, gastric IM is believed to be triggered or accelerated by some type of chronic environmental stimulus, such as *Helicobacter pylori* (*Hp*) infection, bile regurgitation, smoking and alcohol consumption [5, 8]. Although *Hp* infection is considered as the major cause of IM, whether the eradication of *Hp* could stop the development of GC remains controversial [9–11]. Therefore, other important factors may promote the occurrence of IM and GC. Reports have suggested that bile acids (BA) may have the potential to promote the transformation of cells and act as a tumor inducement in gastrointestinal neoplasia [12, 13]. Moreover, a high concentration of BA may promote the development of IM at the gastroesophageal junction of mice [14], and increases the extent and severity of IM is more serious in patients with high concentration of BA [15]. Therefore, additional attention should be focused on the pathogenic mechanisms of BA-induced gastric IM.

Caudal-related homeobox transcription factor 2 (CDX2) is an essential intestine-specific transcription factor (TF) [16, 17] that can modulate the transdifferentiation of IM by stimulating the transcription of intestinal markers, such as MUCIN 2 (MUC2), Krüppel-like factor 4 (KLF4), sucrase isomaltase (S-I) and VILLIN1 (VIL1) [18–20]. In recent years, researchers have focused on elucidating the regulatory mechanisms underlying the abnormal expression of these markers in the process of IM. Noticeably, hepatocyte nuclear factor-4α (HNF4α), which is a nuclear transcription factor highly expressed in the intestine that participates in regulating the development and function of the intestine, has recently been found to be associated with IM [21, 22]. In particular, Kojima et al. [23] found that HNF4α and MUC2 were overexpressed in IM and intestinal GC and are positively correlated. Similarly, we proved that the appearance of gastric IM was promoted by the transcriptional activation function of HNF4α on CDX2 after treatment with BA in vitro (manuscript submitted for publication). However, the activation mechanism of BA on HNF4α is not clear.

In this study, we found that BA caused a significant increase in histone deacetylase 6 (HDAC6) in the gastric cell line GES-1 through RNA-seq. Acetylation and deacetylation of histones are important epigenetic modifications that modulate gene transcription and expression. HDAC6, a special member of the HDAC family, not only participates in histone deacetylation but also acts on some nonhistone substrates to regulate cell metastasis, proliferation, and invasion in tumors [24, 25]. In addition, our previous study revealed that miR-1 was obviously decreased in a BA-induced gastric IM cell model [26]. Interestingly, we predicted through the mirWalk2.0 (https://zmf.umm.uni-heidelberg.de/apps/zmf/mirwalk2/), PicTar (https://pictar.mdc-berlin.de/) and Target scan (https://www.targetscan.org/vert_72/) databases that histone deacetylase 6 (HDAC6) had the same putative miR-1 3′-UTR binding sites as HNF4α. Therefore, we explored the relationship among miR-1, HDAC6 and HNF4α as well as their roles in BA-induced IM.

This study showed that BA led to an increase in HDAC6 and HNF4α by reducing the level of miR-1 and that the two proteins stimulated each other to form a positive loop and eventually caused gastric IM. Based on our results and relevant findings, we hypothesized that the loop regulates the molecular changes that occur with chronic bile regurgitation in gastric mucosa and promotes the progression of IM.

## Materials and methods

### Cell lines

GES-1, an immortalized gastric epithelial cell line, was used mainly to establish the IM model which was described previously [26]. AGS, MKN45 and AZ521 are gastric cancer cell lines. HCT-116 is one kind of colon cancer cell line. All of the cell lines were purchased from ATCC and resuscitated within 6 months and they were also tested negative for mycoplasma contamination. Gastric cells were cultured in PRIM-1640 medium (Gibco, US), while colon cells were cultured in DMEM medium (Gibco, US) and all cell lines were cultured with 10% fetal bovine serum (Biological Industries, Israel), 100 mg/ml streptomycin and 100 U/ml penicillin. Deoxycholic acid (DCA) is a major hydrophobic BA with strong cytotoxicity and it was purchased from BiocytoSci (USA).

### Tissue microarrays and human gastric IM samples

A normal tissue microarray containing 24 cases (BN01011b) and a gastric IM tissue microarray (ST8017a) containing 80 cases were purchased from Alenabio Biotech (China). In addition, 24 normal cases, 15 gastritis cases, and 39 gastric IM cases were also included. Ten paired gastric IM specimens were obtained from patients who underwent endoscopy. To exclude the effect of *Hp* infection, all selected patients were *Hp* negative. All patients signed the informed consent forms before the specimens were obtained. Our study was approved by the Human Subjects Committee of Xijing Hospital. The pathological status of these specimens was collected from the Department of Pathology.

### Hnf4α transgenic mice

The generation of *Lgr5-Cre* and *LSL-Hnf4α* mice were generated on a *C57BL6* background. *Lgr5-Cre* mice were bred with *LSL-Hnf4α* mice to activate *Hnf4α* in gastric cells. To activate *Cre* recombinase in *Rosa26*^*Hnf4α*^ mice, the animals were intraperitoneally injected with 5 mg tamoxifen dissolved in corn oil once a day for three consecutive days at 4 weeks of age. The treatment, maintenance and care of mice in this study followed the protocols of the Animal Research Committee of Xijing Hospital.

### Immunohistochemistry and in situ hybridization

Immunohistochemistry (IHC) was performed using anti-HDAC6 (1:400, Cell Signaling Technology, #7558) and anti-HNF4α (1:100, Abcam, #ab92378) following the manufacturer’s instructions. In situ hybridization (ISH) was carried out by using a 5-digoxigenin (DIG) and 3′-DIG-labeled locked nucleic acid-based probe specific for miR-1 (Exiqon, Denmark) on tissue microarray. The IHC and ISH staining results were independently evaluated by two professional pathologists independently.

### RNA extraction and real-time PCR

TRIzol^®^ reagent (Invitrogen, USA) was used to extract the total RNA from cell lines and human tissue samples according to a standard protocol. Then RNA was reverse-transcribed into cDNA using the PrimeScript^®^ RT Reagent kit (Takara Biotechnology, Japan) and qPCR was conducted using SYBR Premix Ex Taq II (Takara Biotechnology, Japan) on a CFX96™ Real-Time PCR Detection system (Bio-Rad Laboratories, USA). β-Actin and U6 were used as the internal control and the 2^∆∆Cq^ method was used to quantify the relative mRNA expression of each gene. Sequences of gene-specific primers are provided in Table 1.

**Table 1:** Sequence of primers used in the study

Primer name	Forward sequence	Reverse sequence
HDAC6	AGTCCATCGCAGATACTGGC	TTAGTCTGGCCTGGAGTGGA
HNF4α	ACATGGACATGGCCGACTACA	AGCTCGCAGAAAGCTGGGAT
miR-1	TAGAAGCTTGCCTCTGAGCTGCCTTCTCTA	
HDAC6 promoter	AAGATGTTTACTCTCTGTCCC	GTCCGGGGGTTCTGCATCAGAC

### Protein extraction and western blot analysis

Total protein was extracted by mixing cells with a radioimmunoprecipitation assay (RIPA) buffer (Beyotime, China) supplemented with protease and phosphatase inhibitors. Western blot analysis was carried out following standard procedures. The following antibodies were used for this experiment: anti-HDAC6 (1:1000, Cell Signaling Technology, #7558), anti-HNF4α (1:2000, Abcam, #92378), anti-MUC2 (1:4000, Abcam, #ab133555), anti-KLF4 (1:1000, Cell Signaling Technology, #4038), anti-CDX2 (1:1000, Cell Signaling Technology, #12306), and anti-β-actin (1:5000, Bioworld, #AP0060). The primary antibody dilution buffer, PREstain Protein Ladder and Western SuperSensitive Substrate were purchased from BioCytoSci (USA).

### Immunofluorescence cytochemistry

Immunofluorescence (IF) staining for HDAC6 and HNF4α was performed in GES-1 cells. The primary antibodies were a rabbit anti-human HDAC6 antibody (1:200, Cell Signaling Technology, #7558), a rabbit anti-human HNF4α antibody (1:200, Abcam, #92378), a rabbit anti-mouse LGR5 antibody (1:25, Abcam, #75732) and a mouse anti-mouse HNF4α antibody (1:50, Abcam, #374229).

### Transfection

Synthetic miR-1 agomir, antagomir, the corresponding negative control oligonucleotides, plasmid and small interfering RNA (siRNA) were purchased from Genepharma (China), and their sequences are shown in Table 2. The transfection reagent was purchased from Thermo Fisher Scientific (USA) and used following the manufacturer’s protocol. HDAC6 and HNF4α overexpression lentiviral vectors were designed and provided by Genechem Co. Ltd. (China).

**Table 2:** Interference targets of each gene involved in the study

siRNA name	Forward sequence	Reverse sequence
HDAC6	AGTCCATCGCAGATACTGGC	TTAGTCTGGCCTGGAGTGGA
HNF4α	ACATGGACATGGCCGACTACA	AGCTCGCAGAAAGCTGGGAT
miR-1 agomir	UGGAAUGUAAAGAAGUAUGUAU	ACAUACUUCUUUACAUUCCAUU
miR-1 antagomir	AUACAUACUUCUUUACAUUCCA	

### Dual-luciferase report assay

We amplified a 2000 bp sequence upstream of the transcription start sites of the HDAC6 promoter by PCR and then cloned it into a pGL3-basic luciferase vector (Invitrogen) to generate pHDAC6/2000-Luc, which was used to generate HDAC6-p1 (− 2000 to − 1 bp), HDAC6-p2 (− 1589 to − 1 bp), HDAC6-p3 (− 1544 to − 1 bp), HDAC6-p4 (− 1432 to − 1 bp) and HDAC6-p5 (− 1333 to − 1 bp). The 3′-UTR reporter plasmids of HDAC6 and HNF4α for miR-1 were constructed using the chemically synthesized DNA oligos: HNF4α-wtUTR and HDAC6-wtUTR containing the full-length cDNA sequence, and HNF4α-mutUTR and HDAC6-mutUTR expression vectors lacking the 3′-UTR were also constructed.

### Chromatin immunoprecipitation

GES-1 cells were transiently transfected with an HDAC6 promoter vector. Then, chromatin immunoprecipitation (ChIP) analysis was carried out according to the standard method of the Magna ChIP G Assay kit (EMD Millipore. USA). Chromatin was immunoprecipitated with anti-HNF4α (Abcam, #ab181604) or IgG as a negative control. Finally, immunoprecipitated DNA–protein complexes were isolated and a real-time PCR assay was carried out to examine the quantity of the specific proteins. The primers for the HDAC6 promoters are listed in Table 1.

### Statistical analysis

The SPSS software (V.19.0, SPSS, Chicago, Illinois, USA) statistical package was used to conduct all statistical analyses. All continuous data are expressed as the mean ± SD. The *χ*^2^ test was used to compare the frequencies of categorical variables. Mutual associations among clinical results were assessed by using Spearman’s rank correlation. Statistical comparisons between two groups were analyzed with the Mann–Whitney *U* test. Multiple comparisons were performed via a one-way analysis of variance (ANOVA) with the Bonferroni post hoc test. *P* values less than 0.05 were considered statistically significant.

## Results

### HNF4α overexpression in the mouse stomach promotes mucin secretion

**Fig. 1:**
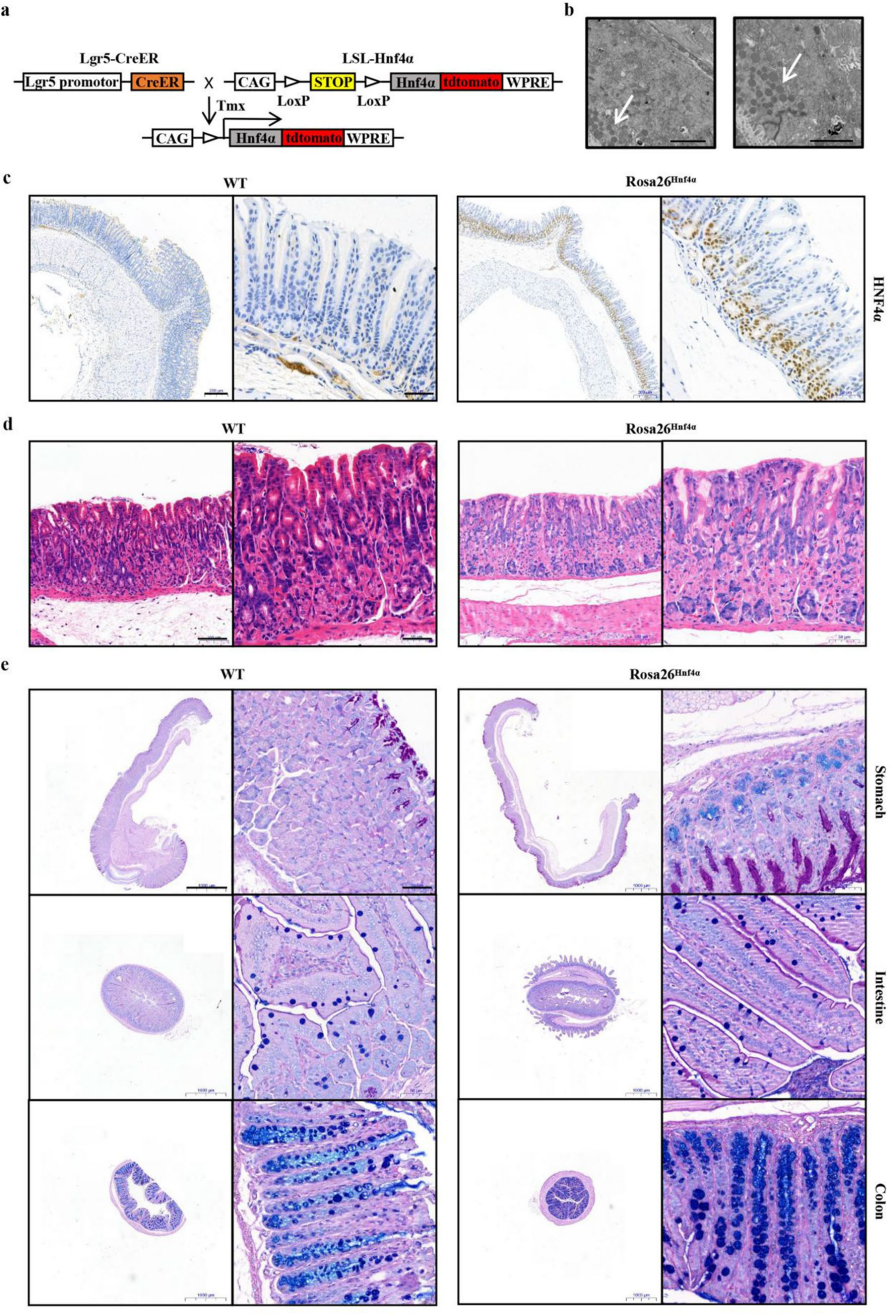
HNF4α-induced gastric mucosal changes in mice. **a** Schematics of the gene constructs for *Lgr5-Cre* mice and *LSL-Hnf4α* mice, and the experimental strategy for Cre-mediated recombination in the *Rosa26*^*Hnf4α*^ mice. **b** Representative electron microscopy pictures showing mucin of *Rosa26*^*Hnf4α*^ mice gastric epithelium (arrows). Scale bar: 2 μm. **c** Immunohistochemistry (IHC) for Hnf4α in the stomach section of *WT* and *Rosa26*^*Hnf4α*^ mice. The right boxes indicate regions enlarged. Scale bars: 200 µm (left); 50 µm (right). **d** HE staining for gastric sections of *WT* and *Rosa26*^*Hnf4α*^ mice. Scale bars: 100 µm. **e** Different sections from *WT* and *Rosa26*^*Hnf4α*^ mice were examined by Alcian blue staining–periodic acid–Schiff (AB–PAS) staining. Scale bars: 1000 µm (left); 50 µm (right)

To better understand the prometaplastic role of Hnf4α in vivo, we constructed an Hnf4α transgenic mouse model that expresses the active form of Hnf4α in Lgr5^+^ gastric stem cells upon tamoxifen exposure (Figs. 1a, S1). The mice were killed and gastric tissues were separated after tamoxifen treatment for 0, 6 and 12 months, respectively. We found that the gastric mucosa of wild-type (WT) mice and *Rosa26*^*Hnf4α*^ mice were both mostly normal at 0 and 6 months (data not shown). Nevertheless, compared with that of WT mice, at 12 months post-tamoxifen, the expression level of Hnf4α in gastric tissues was significantly higher and structural abnormalities were observed in the gastric mucosa of *Rosa26*^*Hnf4α*^ mice (Fig. 1c, d). More importantly, electron microscopy studies revealed that mucin increased in the gastric cells of *Rosa26*^*Hnf4α*^ mice (Fig. 1b). Further, AB-PAS staining showed obvious positive staining in the fovea of gastric mucosa and the bottom of glands in *Rosa26*^*Hnf4α*^ mice, while weak staining was found only in the pits in WT mice (Fig. 1e). Although no obvious IM cells were observed in the gastric mucosa of mice, these changes may still indicate that Hnf4α might affect the development of gastric mucosa and promote the secretion of intestinal mucus.

### HDAC6 is upregulated in BA-induced IM cells

**Fig. 2:**
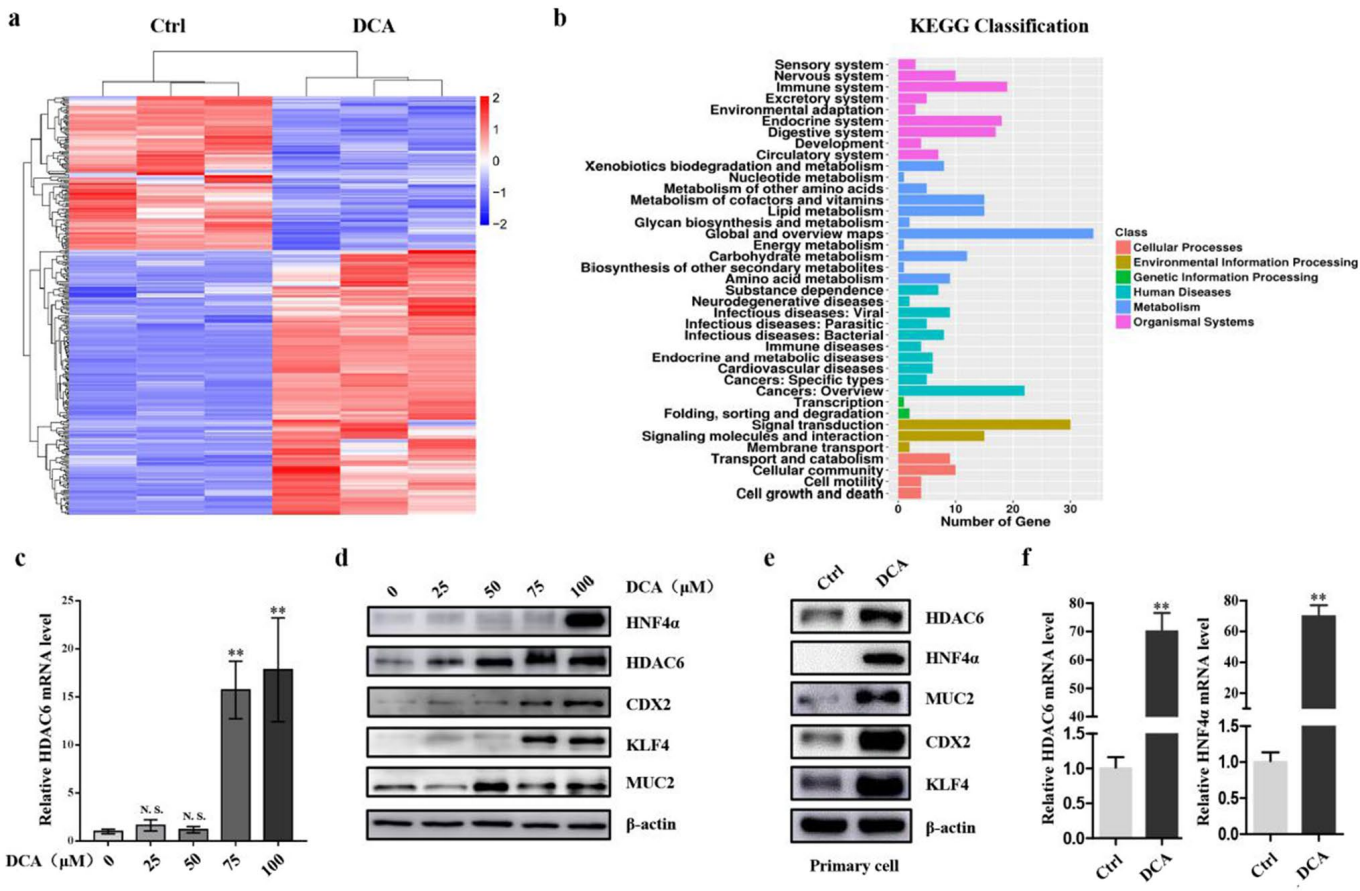
HDAC6 was increased in BA-stimulated IM cells. **a**, **b** Whole genome expression profiles for GES-1 cells treated with DCA. Heat map (left) and KEGG classification (right) illustrating the global differences in gene expression between DCA-treated GES-1 cells and control (fold change > 2.0; *P* < 0.05). Red: increased expression, blue: decreased expression. Incubating time: 24 h; dosage: 100 µM. **c** HDAC6 mRNA level was increased by DCA treatment. Data was normalized to β-actin mRNA. **d** Effects of DCA on the expression of HDAC6, HNF4α and intestinal markers (CDX2, MUC2, KLF4) in GES-1 cells. Cells were stimulated with various concentrations of the DCA or vehicle alone for 24 h, then protein was extracted and subjected to western blot analysis for HDAC6, HNF4α, CDX2, MUC2, KLF4 and β-actin. β-Actin levels were used as internal control in immunoblots. **d, e** DCA (100 µM) enhanced the protein levels of HDAC6, HNF4α and intestinal markers in primary gastric epithelial cells. It also increased the mRNA levels of HDAC6 and HNF4α. *P < 0.05; **P < 0.01. *N.S.* not significant

Initially, we treated GES-1 cells with DCA and extracted RNA for RNA-seq. Figure 2a shows the heat map of differentially expressed molecules, and HDAC6 is one of the significantly increased molecules. KEGG data showed that DCA is closely related to tumor and signal transduction (Fig. 2b). Next, GES-1 cells were treated with a gradient concentration of DCA, and the results showed that the mRNA of HDAC6 and HNF4α increased obviously (Figs. 2c, S2A). We also detected that DCA enhanced the HDAC6 protein along with intestinal markers CDX2, KLF4, MUC2 and HNF4α in GES-1 cells and AZ521 cells (Figs. 2d, S2B). The results of IF further confirmed that DCA caused the enhancement of HDAC6 and HNF4α in GES-1 cells and their expression in HCT-116 cells was used as a positive control (Figure S2C). More importantly, similar results were found in mice primary gastric mucosa cells (Figs. 2e, f). Together, these results suggest that HDAC6 increased significantly in BA-induced IM cells.

### Expression of HDAC6 in IM tissues and its correlation with HNF4α

**Fig. 3:**
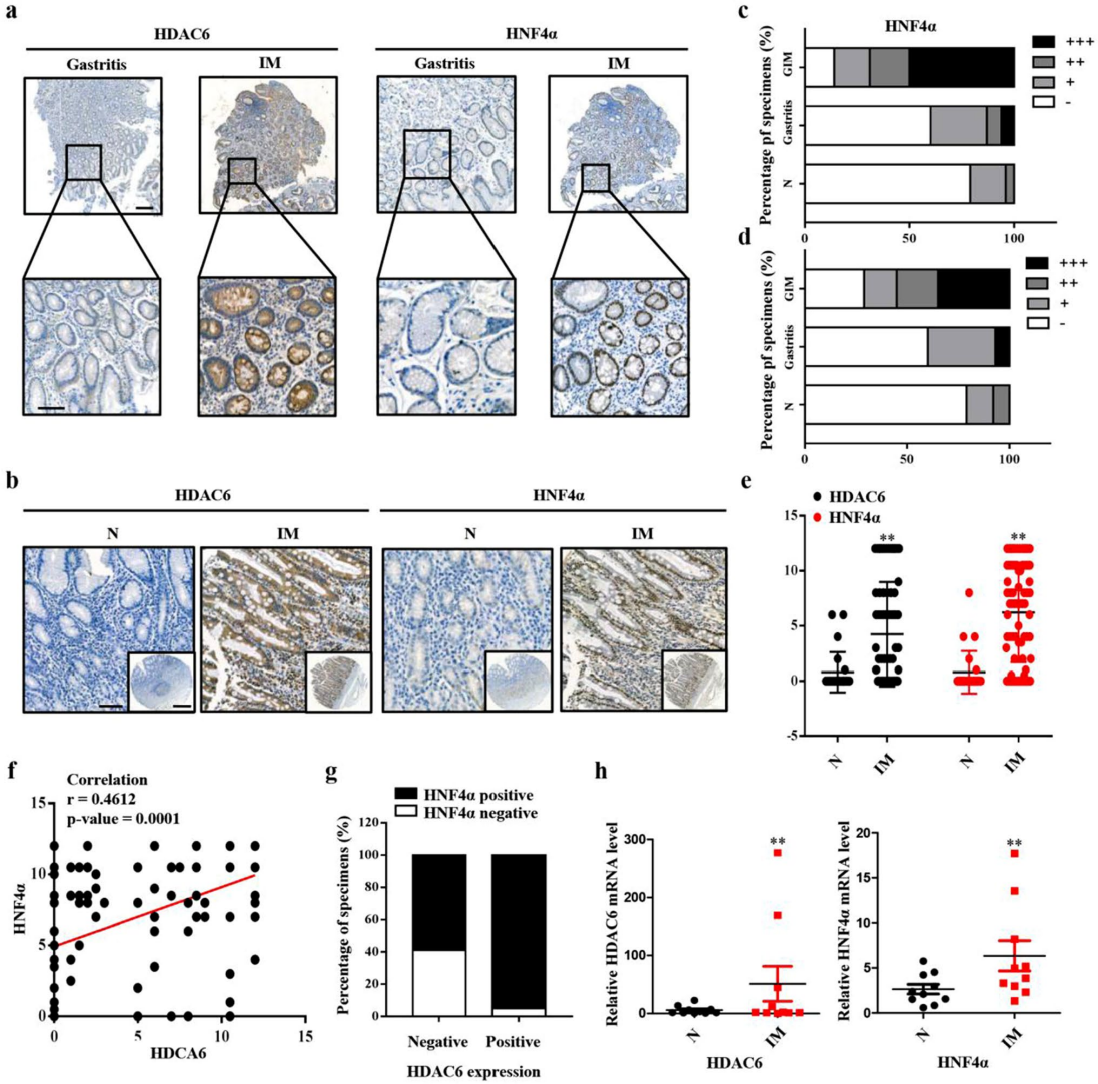
HDAC6 and HNF4α are increased in IM tissues. **a** The representative images of IHC staining for HDAC6 and HNF4α in gastritis and IM tissues. Scale bars: 100 µm (top); 50 µm (bottom). **b** IHC staining for HDAC6 and HNF4α in normal and IM tissues in microarrays. Scale bars: 100 µm; 500 µm (insets). **C, d** Analysis of different expression levels of HDAC6 and HNF4α in normal, gastritis and IM tissues. GIM: gastric intestinal metaplasia. **e** Expression levels of HDAC6 and HNF4α were compared between normal and IM tissues. **f** The correlation between HDAC6 and HNF4α in IM tissue (*r* = 0.4612, *P* = 0.0001). **g** Overlapping of HDAC6 and HNF4α in IM tissues. **h** HDAC6 and HNF4α mRNA levels in ten pairs of matched human IM specimens. Each symbol represents the mean value of an individual patient. **P < 0.01

To investigate the relevance of HDAC6 and HNF4α with gastric IM clinically, we examined the expression of both in normal, gastritis and IM tissues (Fig. 3a, b). Both HDAC6 and HNF4α increased in IM tissues, and the number of samples showing high expression was greater than that showing low or moderate expression in gastric IM tissues (Fig. 3c–e). Additionally, negative expression of HDAC6 and HNF4α were mainly observed in normal tissues, while positive expression was mostly observed in gastric IM tissues (HDAC6: 85/119, 71.4%, *P* = 0.0302; HNF4α: 102/119, 85.7%, *P* = 0.0001). The correlation analysis showed that the expression levels of HDAC6 in IM tissues were positively associated with HNF4α (Fig. 3f and Table 3). Consistently, the staining results of tissue microarrays indicated that most of the HDAC6-positive tissues were also positive for HNF4α. Overlapping expression of the two molecules was seen in 80.67% (96/119) of IM tissues, which further suggested a close correlation between HDAC6 and HNF4α (Fig. 3g and Tables 4, 5). Moreover, as shown in Fig. 3h, the mRNA level of both is higher in IM tissues than in normal tissues. In summary, these findings imply a potential prometaplastic role of HDAC6 and HNF4α in human gastric tissues.

**Table 3:** Correlation between HDAC6 and HNF4α in IM tissues

	HDAC6	HNF4α	Correlation	
			*r*	*p*
Positive	85	102	0.4621	0.0001
Negative	34	17

**Table 4:** HDAC6 expression in normal, gastritis and IM tissues

Histological	*n*	−	HDAC6	Score (*n*)	+++	*p*
			+	++		
Normal	24	19	3	2	0	
Gastritis	15	9	5	0	1	0.0302
IM	119	34	19	24	42

**Table 5:** HNF4α expression in normal, gastritis and IM tissues

Histological	*n*	−	HNF4α	Score (*n*)	+++	*p*
			+	++		
Normal	24	19	4	1	0	
Gastritis	15	9	4	1	1	0.0001
IM	119	17	20	22	60	

### HDAC6 is transcriptionally activated by HNF4α in gastric cells

**Fig. 4:**
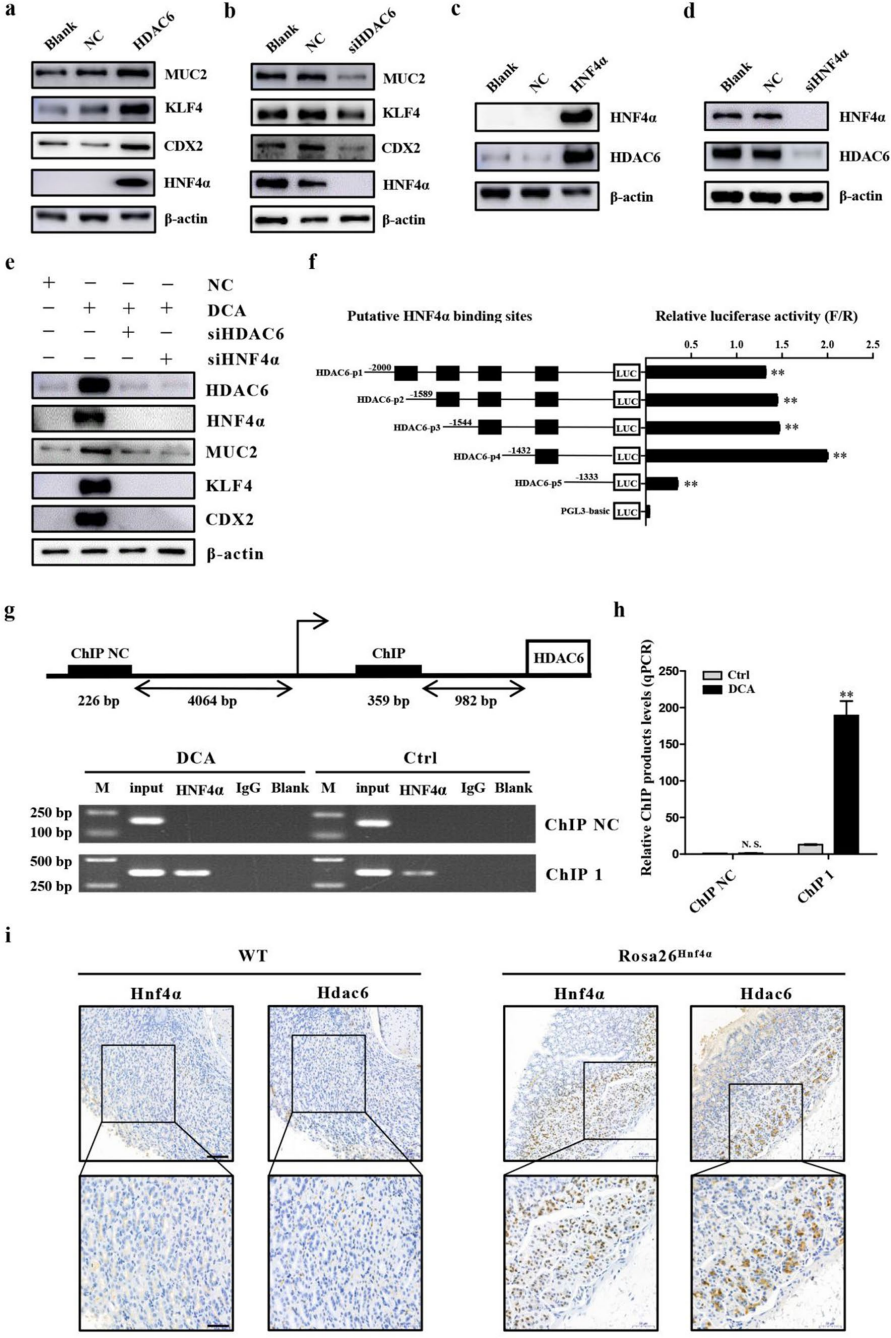
HDAC6 was increased by HNF4α in gastric cells. **a** Western blot analysis showed that overexpression of HDAC6 led to increase of CDX2, KLF4, MUC2 and HNF4α. **b** The protein levels of HNF4α and IM markers were determined by western blot analysis in GES-1 cells transfected with NC or siHDAC6. **c** HDAC6 and HNF4α were measured by western blot in GES-1 cells after infection with HNF4α overexpression vector. **d** Western blot for HDAC6, HNF4α and intestinal markers in GES-1 cells transfected with negative control or siHNF4α. **e** At 24 h after transfection with siHDAC6 or siHNF4α, GES-1 cells were stimulated with the DCA for 24 h. Expression levels of HDAC6, HNF4α and intestinal markers were measured by western blot. Data of each group were compared with the NC group. **f** The truncation reporter genes containing different binding sites were used to analyze the HNF4α transcription activity sites in the HDAC6 promoter region. **g, h** Chromatin immunoprecipitation (ChIP) experiments validated the binding capacity of HNF4α to the HDAC6 promoter. **i** Immunohistochemistry (IHC) for Hnf4α and Hdac6 in the stomach section of *WT* and *Rosa26*^*Hnf4α*^ mice. The below boxes indicate regions enlarged. Scale bars: 100 µm (top); 50 µm (bottom). *P < 0.05; **P < 0.01. *N.S.* not significant

To further study the role of HDAC6 in IM, we performed gain-of-function and loss-of-function experiments. First, we infected GES-1 cells with an HDAC6 overexpression vector, and the results showed that the upregulation of HDAC6 caused an increase of CDX2, KLF4 and MUC2 protein levels (Fig. 4a).Subsequently, AGS cells, which highly express HDAC6 and HNF4α, were transfected with HDAC6-specific siRNA and the results revealed that the downregulation of HDAC6 inhibited the expression of downstream intestinal markers (Fig. 4b). Further, we investigated the regulatory effect of HNF4α on HDAC6 and found that HNF4α overexpression caused a significant increase in HDAC6 in GES-1 cells (Fig. 4c), while siHNF4α resulted in a decrease in HDAC6 in AGS cells, which indicated that HNF4α could positively regulate the expression of HDAC6 in gastric cells (Fig. 4d). Interestingly, HDAC6 was found to also have a positive regulatory effect on HNF4α, which may lead to the formation of a positive feedback regulatory loop. The loop is still effective in the presence of BA as shown in Fig. 4e because both siHDAC6 and siHNF4α could reverse the upregulation of each other and intestinal markers triggered by DCA. Next, to reveal the regulatory mechanism of HNF4α on HDAC6, the JASPAR (https://www.https://jaspar.binf.ku.dk/) and PROMO (https://alggen.lsi.upc.es/cgi-bin/promo_v3/promo/promoinit.cgi?dirDB=TF_8.3) databases were used to predict whether HNF4α had a transcription binding site in the HDAC6 promoter region. Then we constructed the reporter genes for truncated bodies containing different HDAC6 binding sites according to the predicted results of JASPAR and cotransfected them with the HNF4α expression vector in GES cells (Table 1). The data showed that the activity of HDAC6-p4 plasmids increased most obviously, suggesting that the sequence (− 1432 to − 1332 bp) might be the active fragment of HNF4α on the HDAC6 promoter (Fig. 4f). The ChIP assays further verified that HNF4α could combine with the HDAC6 promoter (AGGATCAGAGGGCAA), and this combination was strengthened under DCA (Fig. 4j, h). Additionally, Hdac6 was found to be enhanced by Hnf4α in *Rosa26*^*Hnf4α*^ mice and both were mainly located at the bottom of the gland in the gastric antrum (Fig. 4i). In summary, these results indicated that HDAC6 positively regulated intestinal markers and HNF4α and showed that HNF4α could transcriptionally activate HDAC6 in gastric cells.

### Expression of miR-1 in IM cells

**Fig. 5:**
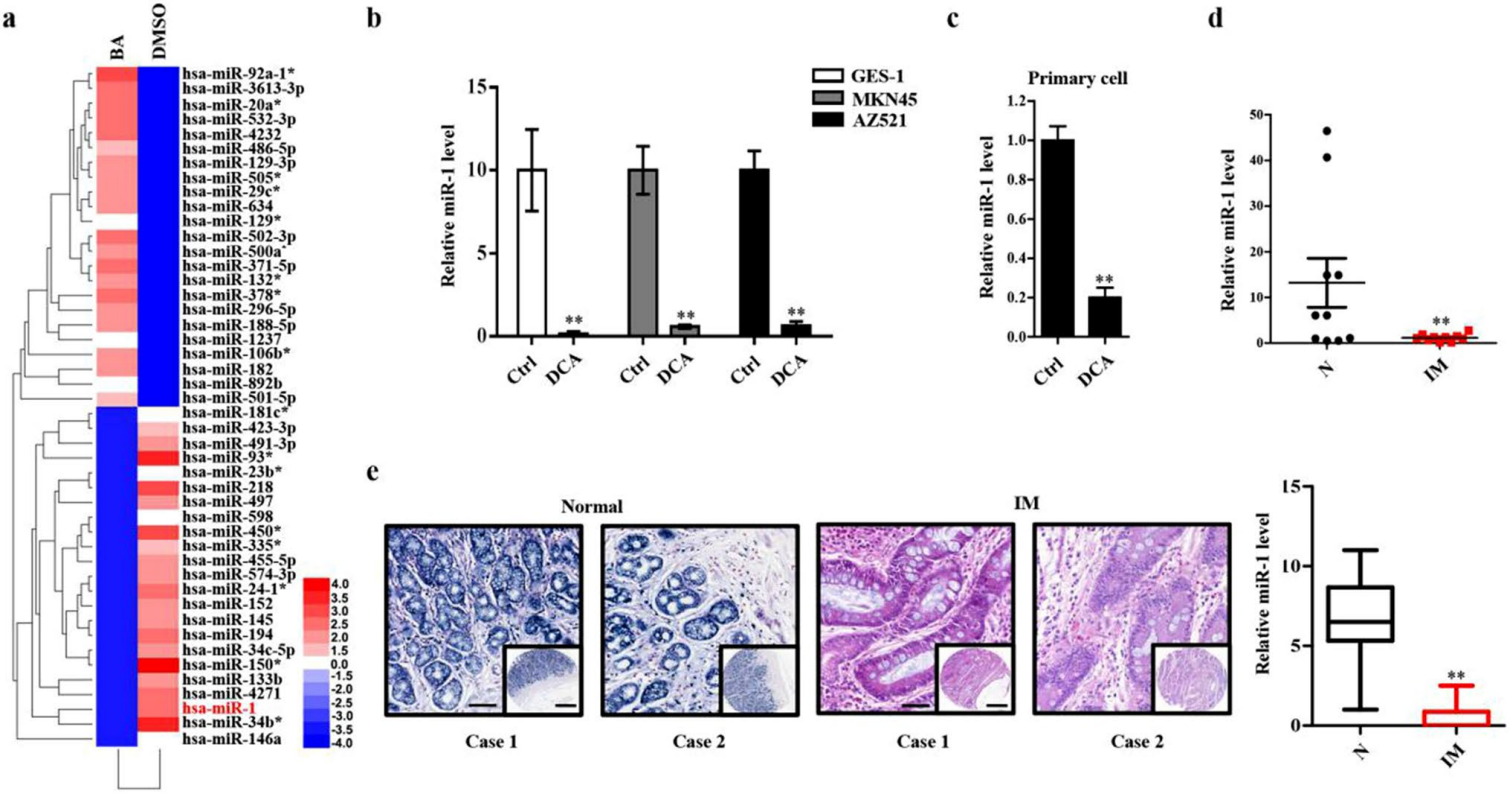
miR-1 was decreased in IM cells. **a** Heatmap of differential microRNA (miRNA) expression between negative control (NC) and BA-treated GES-1 cells reported. Gene expression data were obtained using a human miRNA array. Expression values shown are mean centred. Red: increased expression, blue: decreased expression. Incubating time: 24 h; dosage: 100 µM. **b, c** GES-1, MKN45, AZ521 and mouse primary cells were stimulated with 100 µM DCA or vehicle alone for 24 h, then miR-1 was analyzed by qRT-PCR. U6 was used as an internal control in qRT-PCR of miR-1. **d** The expression levels of miR-1 were compared between normal and IM tissues. **e** Representative images and analysis of in situ hybridization (ISH) staining for miR-1 in normal and IM tissues. Scale bars: 100 µm; 500 µm (insets). *P < 0.05; **P < 0.01. *N.S.* not significant

All the above results indicated that DCA promotes the development of IM through a closed HDAC6/HNF4α loop, although the role of BA in this signaling pathway remains unclear. In a previous study, we sequenced microRNA in GES-1 cells after BA treatment [26]. Combined with a bioinformatics analysis, we found that among several microRNAs that were significantly reduced, miR-1 might be closely related to this loop (Fig. 5a). We speculated that miR-1 might target HDAC6 and HNF4α posttranslationally in IM cells. As expected, the treatment with DCA led to an obvious decrease in miR-1 in GES-1, MKN45 and AZ521 cells (Fig. 5b), and similar results were observed in primary cells post-DCA (Fig. 5c). Furthermore, we found that the level of miR-1 in gastric IM tissues was markedly lower than that in peripheral normal tissues (Fig. 5d). Additionally, microarray in situ hybridization showed that the level of miR-1 in IM was obviously lower than that in normal tissues and the location of miR-1 was in the cytoplasm and nucleus (Fig. 5e). Moreover, the staining indicated that the expression level of miR-1 in IM was remarkably lower than that of HDAC6 or HNF4α and miR-1 was negatively correlated with HDAC6 and HNF4α (Figure S3A, S3B). Collectively, these data suggest that miR-1 decreased in IM tissues and BA-induced IM cells.

### MiR-1 suppresses HDAC6/HNF4α loop and intestinal markers

**Fig. 6:**
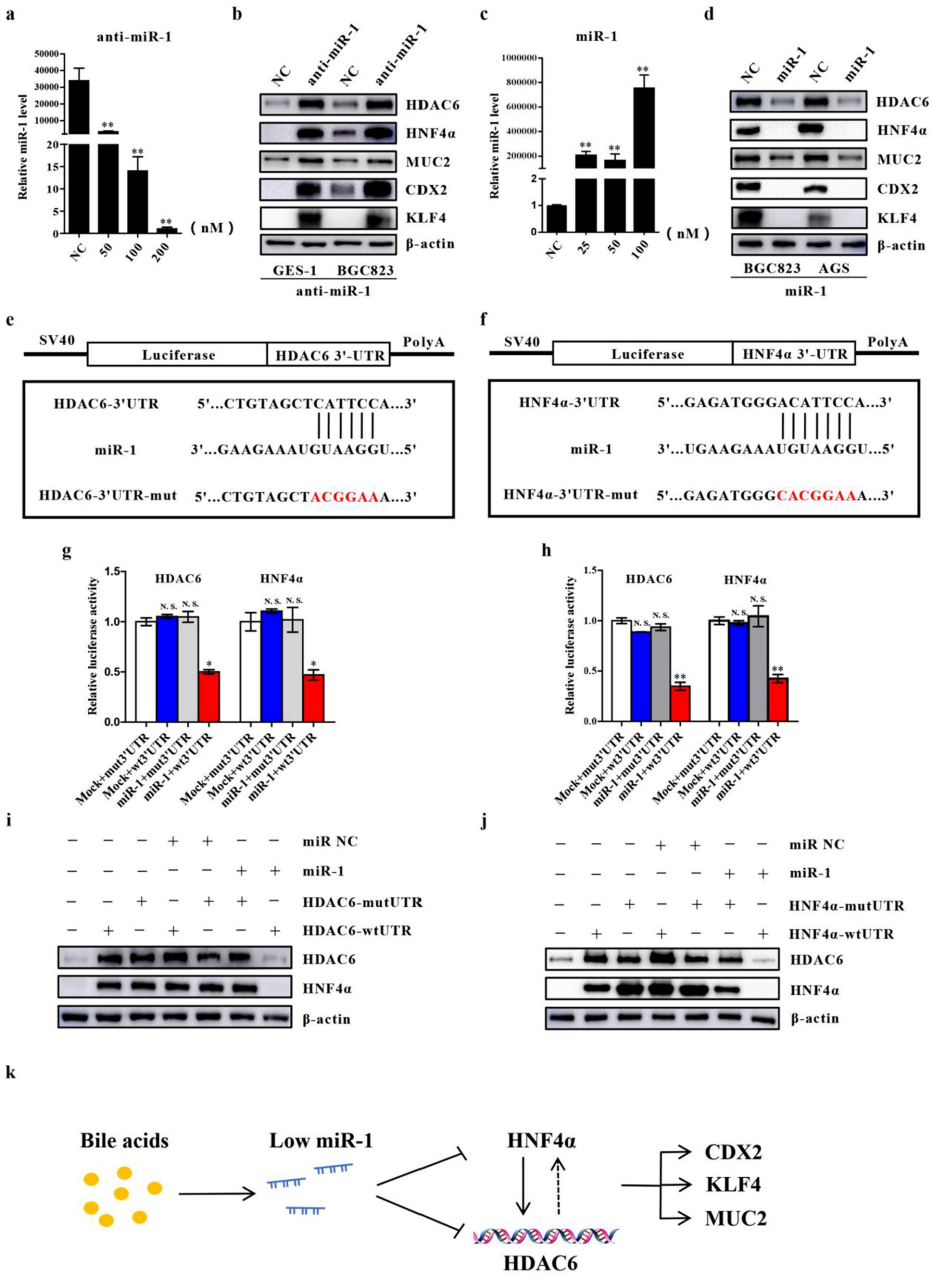
miR-1 downregulated HDAC6 and HNF4α by directly binding its 3′-UTR. **a, b** Downregulated expression of miR-1 led to downregulated protein expression of HDAC, HNF4α and intestinal markers. **c, d** Upregulated expression of miR-1 resulted in the opposite changes. **e, f** A schematic representation of the HDAC6 and HNF4α 3′-UTR. Mutations were generated at the predicted miR-1-binding sites. **g, h** miR-1 luciferase reporter assay. A luciferase reporter was fused with the wild-type or mutant miR-1 targets (HDAC6 and HNF4α), and then transfected into mock-infected or miR-1-infected GES-1 and BGC-823 cells. The luciferase activity of the wild-type luciferase reporters were suppressed by miR-1 significantly. **i, j** GES-1 cells were transfected with wild-type and mutant miR-1 targets (HDAC6, HNF4α), along with miR-1. HDAC6, HNF4α and intestinal markers expression were detected by immunoblots. **k** A schematic model of miR-1/HDAC6/HNF4α pathway in gastric cells. Induced by specific concentrations of bile acid, silenced miR-1 stimulates the expression of HDAC6 and HNF4α to activate downstream intestinal markers. *P < 0.05; **P < 0.01. *N.S.* not significant

To confirm that miR-1 could target HDAC6 and HNF4α, we treated GES-1 cells with a concentration gradient of anti-miR-1. We found that 200 nM anti-miR-1 remarkably reduced the level of miR-1 (Fig. 6a). Then, we used 200 nM anti-miR-1 to transfect GES-1 and BGC-823 cells, and found a robust enhancement of HDAC6 and HNF4α levels accompanied by an increase in intestinal markers (Fig. 6b). Moreover, we transfected AGS and BGC-823 cells with ago-miR-1 and observed that 100 nM ago-miR-1 caused an obvious increase in miR-1 (Fig. 6c). Additionally, HDAC6 and HNF4α decreased substantially with the reduction of CDX2, KLF4 and MUC2 after transfection (Fig. 6d), which is similar to the results from HDAC6 or HNF4α downregulation. These results suggest that the expression of miR-1 could inversely affect the expression levels of HDAC6 and HNF4α. To show that the regulation of the two molecules by miR-1 is achieved by acting on their 3′-UTRs, we carried out luciferase reporter assays (Fig. 6e, 6f). In the GES-1 and BGC-823 cells, the activity of the HDAC6 wt3′-UTR was decreased by miR-1 restoration, while the HDAC6 mut3′-UTR with a mutated binding site sequence was not affected by miR-1. Similarly, the activity of the HNF4α wt3′-UTR was also significantly inhibited by miR-1 (Fig. 6g, h). A quantitative protein analysis showed that HDAC6 levels increased in cells transfected with the *WT* or mutant HDAC6 plasmids, but were attenuated in cells cotransfected with the *WT* HDAC6 and miR-1 (Fig. 6i). Similarly, cotransfection of both HNF4α and miR-1 did not cause an increase in HNF4α (Fig. 6j). In addition, miR-1 attenuated the activation of HDAC6 and HNF4α by DCA (Figure S3C), which indicated that miR-1 is crucial in this process. Our findings demonstrate that the ectopic expression of HDAC6 and HNF4α induced by BA occurs mainly via miR-1 silencing.

## Discussion

This study determined that the BA-induced loop, involving HDAC6 and HNF4α is posttranscriptionally inhibited by miR-1 in BA-induced IM. HNF4α directly targets HDAC6, while miR-1 directly regulates HDAC6 and HNF4α in gastric cells. Hence, the loop which is modulated by miR-1 plays an important role in gastric IM triggered by BA.

At present, the occurrence of IM is believed to involve the abnormal expression of some intestinal markers in gastric mucosa. Reports have indicated that the ectopic expression of CDX2 in the esophagus and gastric mucosa of mice could successfully induce IM [27, 28]. Moreover, BA has been shown to induce the expression of CDX2 in esophageal epithelial cells and then Barrett's esophagus (BE) cells [29]. However, the mechanism underlying the initiation of gastric IM stimulated by BA is still unclear. Previously, we observed that BA could increase CDX2 and induce the IM phenotype through the miR-92a/FOXD1/NF-κB axis, SOX2/CDX2 complex or FXR/SHP axis in GES-1 cells [28, 30, 31]. Herein, we further confirmed in vivo that HNF4α can cause an abnormal morphology of gastric mucosa and led to the secretion of intestinal mucus and we also found that it could interact with HDAC6 to promote the expression of intestinal markers. However, our understanding of aberrant HNF4α expression in IM cells remains unclear.

The miRNA array showed that BA remarkably inhibits miR-1, and the miR-1 sequence is bioinformatically predicted to bind to the 3′-UTR of HNF4α. Similarly, miR221/222 and miR-92a are upregulated by BA in esophagus cells and in gastric cells, respectively, suggesting that miRNAs may serve as an inevitable role in BA-triggered IM [32]. However, direct evidence of the relationship between miR-1 and IM or BA is lacking. HDAC6 has been predicted to have the same binding sequence to miR-1 as HNF4α. We showed for the first time that silencing miR-1 in gastric cells led to the activation of the two targets and the downstream intestinal markers. Once the level of miR-1 was negatively regulated by BA, the loop will be triggered and continue to occur.

Numerous studies have found that HDAC6 could stimulate tumor progression by promoting tumor cell transformation [33, 34], thereby facilitating tumor proliferation [35–37], and regulating tumor immunity [38–41]. In addition, HDAC6 was dramatically increased in *Hp-*positive gastric IM and GC tissues, suggesting that it may have a definite correlation with IM [42]. Sandeep Akare et al. discovered that BA could maintain chromosome stability, and they stimulated cell differentiation by inducing HDAC6 expression in colon cancer cells, which implies that there is a correlation between BA and HDAC6 [43]. Our study not only reconfirmed the activation of BA on HDAC6, but also clarified the prometaplastic function of HDAC6 in BA-induced gastric IM. In this process, HNF4α binds to the HDAC6 promoter and activates its transcription. Moreover, HDAC6 could stimulate the expression of HNF4α to form a closed loop.

We also attempted to determine whether DCA augmented HDAC6 and HNF4α expression in primary gastric cells of mice, and the results were similar to that in gastric cell lines. Above all, our mouse model reaffirmed that HNF4α could augment HDAC6 expression and showed that HDAC6 and HNF4α were both responsible for the induction of mucin secretion in gastric cells. However, typical intestinal metaplasia cells were not observed in the gastric mucosa of transgenic mice, and we will continue to observe the changes of gastric mucosa in subsequent experiments. Moreover, the effects of BA on HDAC6 and HNF4α have not been tested in vivo; thus further studies are required. Despite the deficiencies, this study clearly indicated that forced expression of HNF4α and HDAC6 in gastric epithelial cells promotes a series of molecular changes, followed by the appearance of IM cells. These findings are helpful for better understanding the underlying mechanisms of BA-induced gastric IM. Collectively, our findings demonstrate for the first time that BA causes the high expression of HDAC6 and HNF4α as well as the low expression of miR-1 in gastric cells (Fig. 6k) and both HDAC6 and HNF4α could facilitate the expression of each other. More importantly, miR-1, which is downregulated in IM cells, inhibited both HDAC6 and HNF4α by directly binding to their 3′-UTR.

In summary, our study revealed a new HDAC6/HNF4α loop regulated by miR-1, which is helpful for further elucidating the underlying mechanism of gastric IM. Suppression of the HDAC6/HNF4α loop and restoration of miR-1 may be a promising approach for gastric IM in patients with bile reflux.

## Electronic supplementary material

Below is the link to the electronic supplementary material.

Supplementary file1 (PDF 579 kb)

## Data Availability

All data generated or analyzed during this study are included in this manuscript.

## References

[1] Bray F, Ferlay J, Soerjomataram I, Siegel RL, Torre LA, Jemal A (2018). Global cancer statistics 2018: GLOBOCAN estimates of incidence and mortality worldwide for 36 cancers in 185 countries. CA Cancer J Clin.

[2] Sue S, Shibata W, Maeda S (2015). Helicobacter pylori-induced signaling pathways contribute to intestinal metaplasia and gastric carcinogenesis. Biomed Res Int.

[3] Correa P (1992). Human gastric carcinogenesis: a multistep and multifactorial process–first American Cancer Society Award Lecture on Cancer Epidemiology and Prevention. Cancer Res.

[4] Uemura N, Okamoto S, Yamamoto S, Matsumura N, Yamaguchi S, Yamakido M (2001). Helicobacter pylori infection and the development of gastric cancer. N Engl J Med.

[5] Li D, Bautista MC, Jiang SF, Daryani P, Brackett M, Armstrong MA (2016). Risks and predictors of gastric adenocarcinoma in patients with gastric intestinal metaplasia and dysplasia: a population-based study. Am J Gastroenterol.

[6] de Vries AC, Meijer GA, Looman CW, Casparie MK, Hansen BE, van Grieken NC (2007). Epidemiological trends of pre-malignant gastric lesions: a long-term nationwide study in the Netherlands. Gut.

[7] de Vries AC, van Grieken NC, Looman CW, Casparie MK, de Vries E, Meijer GA (2008). Gastric cancer risk in patients with premalignant gastric lesions: a nationwide cohort study in the Netherlands. Gastroenterology.

[8] Choi AY, Strate LL, Fix MC, Schmidt RA, Ende AR, Yeh MM (2018). Association of gastric intestinal metaplasia and East Asian ethnicity with the risk of gastric adenocarcinoma in a U.S. population. Gastrointest Endosc..

[9] Jiang JX, Liu Q, Zhao B, Zhang HH, Sang HM, Djaleel SM (2017). Risk factors for intestinal metaplasia in a southeastern Chinese population: an analysis of 28,745 cases. J Cancer Res Clin Oncol.

[10] Leung WK, Lin SR, Ching JY, To KF, Ng EK, Chan FK (2004). Factors predicting progression of gastric intestinal metaplasia: results of a randomised trial on Helicobacter pylori eradication. Gut.

[11] Lee YC, Chen TH, Chiu HM, Shun CT, Chiang H, Liu TY (2013). The benefit of mass eradication of Helicobacter pylori infection: a community-based study of gastric cancer prevention. Gut.

[12] Reddy BS, Sharma C, Simi B, Engle A, Laakso K, Puska P (1987). Metabolic epidemiology of colon cancer: effect of dietary fiber on fecal mutagens and bile acids in healthy subjects. Cancer Res.

[13] Kaur BS, Ouatu-Lascar R, Omary MB, Triadafilopoulos G (2000). Bile salts induce or blunt cell proliferation in Barrett's esophagus in an acid-dependent fashion. Am J Physiol Gastrointest Liver Physiol.

[14] Debruyne PR, Witek M, Gong L, Birbe R, Chervoneva I, Jin T (2006). Bile acids induce ectopic expression of intestinal guanylyl cyclase C Through nuclear factor-kappaB and Cdx2 in human esophageal cells. Gastroenterology.

[15] Tatsugami M, Ito M, Tanaka S, Yoshihara M, Matsui H, Haruma K (2012). Bile acid promotes intestinal metaplasia and gastric carcinogenesis. Cancer Epidemiol Biomark Prev.

[16] Silberg DG, Swain GP, Suh ER, Traber PG (2000). Cdx1 and cdx2 expression during intestinal development. Gastroenterology.

[17] Guo RJ, Suh ER, Lynch JP (2004). The role of Cdx proteins in intestinal development and cancer. Cancer Biol Ther.

[18] Barros R, Freund JN, David L, Almeida R (2012). Gastric intestinal metaplasia revisited: function and regulation of CDX2. Trends Mol Med.

[19] Reis CA, David L, Correa P, Carneiro F, de Bolos C, Garcia E (1999). Intestinal metaplasia of human stomach displays distinct patterns of mucin (MUC1, MUC2, MUC5AC, and MUC6) expression. Cancer Res.

[20] Furumiya M, Inoue K, Ohta K, Hayashi Y, Yuasa H (2013). Transcriptional regulation of PCFT by KLF4, HNF4alpha, CDX2 and C/EBPalpha: implication in its site-specific expression in the small intestine. Biochem Biophys Res Commun.

[21] Torres-Padilla ME, Fougere-Deschatrette C, Weiss MC (2001). Expression of HNF4alpha isoforms in mouse liver development is regulated by sequential promoter usage and constitutive 3' end splicing. Mech Dev.

[22] Watt AJ, Garrison WD, Duncan SA (2003). HNF4: a central regulator of hepatocyte differentiation and function. Hepatology.

[23] Kojima K, Kishimoto T, Nagai Y, Tanizawa T, Nakatani Y, Miyazaki M (2006). The expression of hepatocyte nuclear factor-4alpha, a developmental regulator of visceral endoderm, correlates with the intestinal phenotype of gastric adenocarcinomas. Pathology.

[24] Zhang X, Yuan Z, Zhang Y, Yong S, Salas-Burgos A, Koomen J (2007). HDAC6 modulates cell motility by altering the acetylation level of cortactin. MolCell.

[25] Aoyagi S, Archer TK (2005). Modulating molecular chaperone Hsp90 functions through reversible acetylation. Trends Cell Biol.

[26] Li T, Guo H, Li H, Jiang Y, Zhuang K, Lei C (2019). MicroRNA-92a-1-5p increases CDX2 by targeting FOXD1 in bile acids-induced gastric intestinal metaplasia. Gut.

[27] Silberg DG, Sullivan J, Kang E, Swain GP, Moffett J, Sund NJ (2002). Cdx2 ectopic expression induces gastric intestinal metaplasia in transgenic mice. Gastroenterology.

[28] Shi XY, Bhagwandeen B, Leong AS (2008). CDX2 and villin are useful markers of intestinal metaplasia in the diagnosis of Barrett esophagus. Am J Clin Pathol.

[29] Kazumori H, Ishihara S, Rumi MA, Kadowaki Y, Kinoshita Y (2006). Bile acids directly augment caudal related homeobox gene Cdx2 expression in oesophageal keratinocytes in Barrett's epithelium. Gut.

[30] Yuan T, Ni Z, Han C, Min Y, Sun N, Liu C (2019). SOX2 interferes with the function of CDX2 in bile acid-induced gastric intestinal metaplasia. Cancer Cell Int.

[31] Zhou H, Ni Z, Li T, Su L, Zhang L, Liu N (2018). Activation of FXR promotes intestinal metaplasia of gastric cells via SHP-dependent upregulation of the expression of CDX2. Oncol Lett.

[32] Matsuzaki J, Suzuki H, Tsugawa H, Watanabe M, Hossain S, Arai E (2013). Bile acids increase levels of microRNAs 221 and 222, leading to degradation of CDX2 during esophageal carcinogenesis. Gastroenterology.

[33] Salemi LM, Maitland M, Yefet ER, Schild-Poulter C (2017). Inhibition of HDAC6 activity through interaction with RanBPM and its associated CTLH complex. BMC Cancer.

[34] Putcha P, Yu J, Rodriguez-Barrueco R, Saucedo-Cuevas L, Villagrasa P, Murga-Penas E (2015). HDAC6 activity is a non-oncogene addiction hub for inflammatory breast cancers. Breast Cancer Res.

[35] Li D, Sun X, Zhang L, Yan B, Xie S, Liu R (2014). Histone deacetylase 6 and cytoplasmic linker protein 170 function together to regulate the motility of pancreatic cancer cells. Protein Cell.

[36] Lee YS, Lim KH, Guo X, Kawaguchi Y, Gao Y, Barrientos T (2008). The cytoplasmic deacetylase HDAC6 is required for efficient oncogenic tumorigenesis. Cancer Res.

[37] Witt O, Deubzer HE, Milde T, Oehme I (2009). HDAC family: what are the cancer relevant targets?. Cancer Lett.

[38] Cheng F, Lienlaf M, Perez-Villarroel P, Wang HW, Lee C, Woan K (2014). Divergent roles of histone deacetylase 6 (HDAC6) and histone deacetylase 11 (HDAC11) on the transcriptional regulation of IL10 in antigen presenting cells. Mol Immunol.

[39] Tumeh PC, Harview CL, Yearley JH, Shintaku IP, Taylor EJ, Robert L (2014). PD-1 blockade induces responses by inhibiting adaptive immune resistance. Nature.

[40] Shimizu T, Seto T, Hirai F, Takenoyama M, Nosaki K, Tsurutani J (2016). Phase 1 study of pembrolizumab (MK-3475; anti-PD-1 monoclonal antibody) in Japanese patients with advanced solid tumors. Invest New Drugs.

[41] Hao M, Zhao G, Du X, Yang Y, Yang J (2016). Clinical characteristics and prognostic indicators for metastatic melanoma: data from 446 patients in north China. Tumour Biol.

[42] Wang F, Luo LD, Pan JH, Huang LH, Lv HW, Guo Q (2012). Comparative genomic study of gastric epithelial cells co-cultured with Helicobacter pylori. World J Gastroenterol.

[43] Akare S, Jean-Louis S, Chen W, Wood DJ, Powell AA, Martinez JD (2006). Ursodeoxycholic acid modulates histone acetylation and induces differentiation and senescence. Int J Cancer.

