# Supplementary material for: HDAC6/HNF4α loop mediated by miR-1 promotes bile acids-induced gastric intestinal metaplasia
Source: Gastric Cancer. 2020 Jul 23;24(1):103–16. doi: 10.1007/s10120-020-01108-x (PMC7790803; doi:10.1007/s10120-020-01108-x)
Supplement: Supplementary file 1 — Supplementary file1 (PDF 579 kb) [file 10120_2020_1108_MOESM1_ESM.pdf]

Supplementary Figures

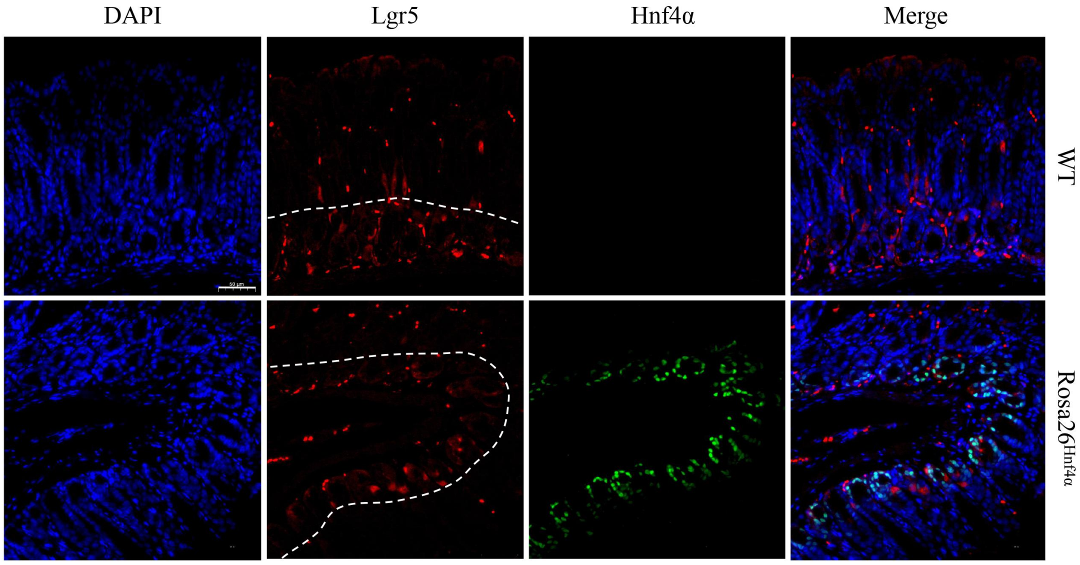

Figure S1. Lgr5Cre-mediated active Hnf4α expression in murine gastric Lgr5+ stem cells. Immunofluores-cence (IF) analysis using DAPI (blue), Lgr5 (red, for Lgr5+ cells) and Hnf4α (green) antibodies in Lgr5+ stem cells from *WT* and $Rosa26^{Hnf4\alpha}$ antrum 1 day after tamoxifen (Tam) administration. The glandular base of the mucosa is distinguished from the rest of the mucosa by a white dashed line. Scale bars: 50 µm.

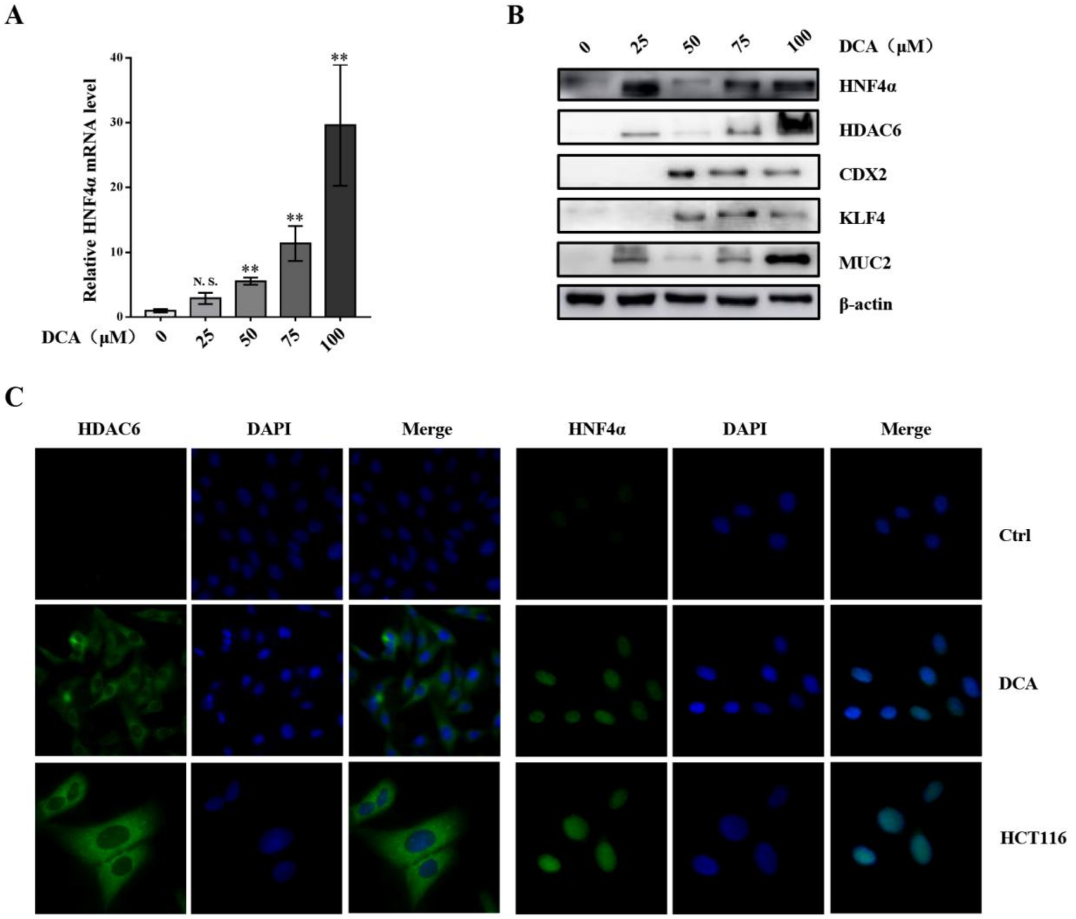

Figure S2. DCA induces HDAC6 and HNF4α expression. (**A**) HNF4α mRNA level was increased by DCA treatment. (**B**) Effects of gradient concentration of DCA on the expression of HDAC6, HNF4α and intestinal markers (CDX2, MUC2, KLF4) in AZ521 cells. (**C**) Effects of DCA on HDAC6 and HNF4α protein expressions in GES-1 cells was determined by immunofluorescence. After incubation with 100μM of the DCA for 24 hours, HDAC6 and HNF4α positive cells with nuclear staining (DAPI) were observed. HCT116 as a positive control. Representative results of three separate experiments are shown. *p<0.05; **p<0.01. N.S., not significant.

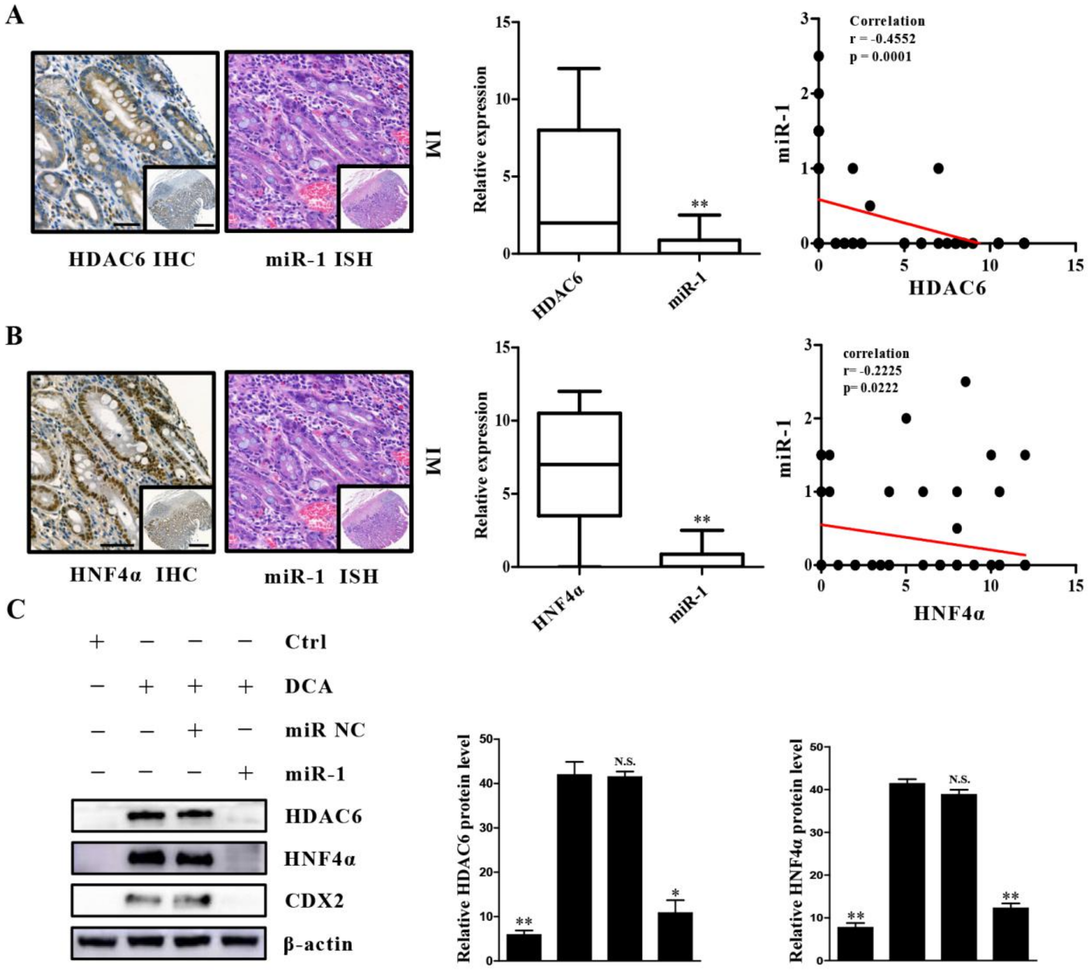

Figure S3. MiR-1 inhibits HDAC6 and HNF4α activity by targeting their 3'UTR. IHC and ISH results showed that expression level of HDAC6 (**A**) and HNF4α (**B**) was negatively associated with miR-1 in IM tissues. Scale bars: 100 μm; 500 μm (insets). (**C**) Upregulation of miR-1 resulted in downregulated protein expression of HDAC6, HNF4α and CDX2 in GES-1 cells after DCA. *p<0.05; **p<0.01. N.S., not significant.
